# Putative risk and resiliency factors among Royal Canadian Mounted Police cadets

**DOI:** 10.3389/fpsyg.2023.1048573

**Published:** 2023-03-15

**Authors:** Juliana M. B. Khoury, Laleh Jamshidi, Robyn E. Shields, Jolan Nisbet, Tracie O. Afifi, Amber J. Fletcher, Sherry H. Stewart, Gordon J. G. Asmundson, Shannon Sauer-Zavala, Gregory P. Krätzig, R. Nicholas Carleton

**Affiliations:** ^1^Canadian Institute for Public Safety Research and Treatment-Institut Canadien de recherche et de traitement en sécurité publique (CIPSRT-ICRTSP), University of Regina/Université de Regina, Regina, SK, Canada; ^2^Anxiety and Illness Behaviours Laboratory, Department of Psychology, University of Regina, Regina, SK, Canada; ^3^Department of Community Health Sciences, University of Manitoba, Winnipeg, MB, Canada; ^4^Department of Sociology and Social Studies, University of Regina, Regina, SK, Canada; ^5^Mood, Anxiety, and Addiction Comorbidity (MAAC) Lab, Departments of Psychiatry and Psychology and Neuroscience, Dalhousie University, Halifax, NS, Canada; ^6^Treatment Innovation for Psychological Services Research Program, Department of Psychology, University of Kentucky, Lexington, KY, United States; ^7^Department of Psychology, University of Regina, Regina, SK, Canada

**Keywords:** public safety personnel, RCMP cadets, mental health, putative risk factors, resiliency factors

## Abstract

**Objective:**

Mental health disorders are prevalent among active-duty Royal Canadian Mounted Police (RCMP) officers. The current study was designed to assess whether RCMP cadets commencing the Cadet Training Program are inherently at greater risk of developing mental health challenges by statistically comparing cadet putative risk and resiliency scores to scores from young adult populations. The study was also designed to assess for sociodemographic differences in putative risk and resiliency variables among RCMP cadets in order to facilitate future comparisons.

**Methods:**

Cadets (*n* = 772; 72.2% men) completed self-report measures of several putative risk variables (i.e., anxiety sensitivity, fear of negative evaluation, pain anxiety, illness and injury sensitivity, intolerance of uncertainty, and state anger) and resiliency. Scores were statistically compared to samples from Canadian, American, Australian, and European young adult populations.

**Results:**

Cadets had statistically significantly lower scores on all putative risk variables and statistically significantly higher resiliency scores compared to the young adult populations. In the cadet sample, there were statistically significant differences in putative risk and resiliency variables across gender and sex.

**Conclusion:**

Cadets’ significantly lower scores on putative risk variables and higher scores on resiliency suggest that they may be psychologically strong; as such, it may be that the nature of police work, as opposed to inherent individual differences in risk and resiliency, accounts for active-duty RCMP officers’ comparatively higher prevalence of mental health disorders over time.

**Clinical Trial Registration**: ClinicalTrials.gov, Identifier NCT05527509.

## Introduction

Public safety personnel (PSP; e.g., border services agents, correctional workers, firefighters, paramedics, police officers, and public safety communicators) work to support the safety of citizens (Canadian Institute of Public Safety Research and Treatment, 2019; Government of Canada, 2019); accordingly, PSP are frequently exposed to diverse potentially psychologically traumatic events (PPTE; Carleton et al., 2019). PPTE have been associated with increased reports of mental health symptoms in Canadian PSP (Carleton et al., 2019). Approximately 50% of Royal Canadian Mounted Police (RCMP) officers screen positive for one or more mental health disorders (Carleton et al., 2018), which is much higher than the screening prevalence of 10.1% in the Canadian general population (Government of Canada, 2012).

Since World War II, there have been suggestions that these elevated rates of mental health challenges among uniformed service personnel are due to inherent weaknesses that could be screened for prior to service, rather than a result of the pressures of PPTE exposures on the job (Horswill and Carleton, 2021). Personality screens, such as the Woodworth Test, Army Alpha, and Army Beta, were used unsuccessfully during the World Wars to identify soldiers inclined to develop psychological problems from battle exposure (Horswill and Carleton, 2021). Shell Shock and hysteria were recognized as legitimate diagnoses resulting from exposure to war but soldiers with such conditions were considered inherently psychologically weak persons who would have still experienced mental health challenges had they remained civilians (Duguid, 1938; Horswill and Carleton, 2021). Remnants of such thinking persist today regarding PSP (Burns and Buchanan, 2020; Ricciardelli et al., 2020a), as evidenced by cultures of stoicism in policing organizations (McElheran and Stelnicki, 2021) and the use of personality screening measures with police recruits not only to assess for vocational goodness of fit, but also for the potentiality for psychological problems (Pozzulo et al., 2017). Nevertheless, the ability of personality screens to predict mental health outcomes among police appears modest at best (Detrick et al., 2001; Koepfler et al., 2012).

Research on putative risk factors for mental health disorders has increasingly focused on individual difference variables (e.g., Barlow et al., 2004; Paulus et al., 2015), including anxiety sensitivity, intolerance of uncertainty, fear of negative evaluation, illness and injury sensitivity, pain-related anxiety, state anger, and self-reported resilience. While some argue that some of these putative risk factors are personality factors (e.g., anxiety sensitivity, fear of negative evaluation), they are relatively more modifiable than more commonly agreed upon personality traits like neuroticism and extraversion (e.g., Smits et al., 2008; Keough and Schmidt, 2012). These individual difference variables are associated with diverse mental health challenges both in the general population (Schmidt and Lerew, 1998; Schmidt et al., 1999; O'Connor et al., 2002; Asmundson and Stapleton, 2008; Meffert et al., 2008; Capron et al., 2012; Carleton et al., 2012a; Van der Molen et al., 2014; Carleton, 2016; de Bles et al., 2019; Korol et al., 2019; Zegel et al., 2022) and among PSP (Feeny et al., 2000; Asmundson and Stapleton, 2008; Meffert et al., 2008; Carleton et al., 2009, 2018; Boffa et al., 2018; Stanley et al., 2018; de Bles et al., 2019; Korol et al., 2019; Rogers et al., 2020; Zegel et al., 2022). Reducing individual risk factors may necessarily increase individual resilience but there is also literature suggesting that resilience is an independent individual difference variable broadly defined as the ability to successfully adapt to difficult situations (Luthar, 2000). Accordingly, resilience correlates negatively with mental health challenges (Smith et al., 2008; Rodríguez-Rey et al., 2016; Sánchez et al., 2021). Among PSP, higher levels of self-reported resilience are associated with lower levels of symptoms related to PTSD and other mental health disorders (e.g., Smith et al., 2008; Lee et al., 2014; Kyriazos et al., 2018; Russell et al., 2021).

The current study is the first attempt, to our knowledge, to evaluate putative risk and resiliency factors among RCMP cadets. Given that results of prior research have shown that RCMP officers have a high prevalence of mental health challenges (Carleton et al., 2018), examining pre-service data can help elucidate whether such challenges stem from inherent individual differences and/or from the nature of service. The current study was designed to assess self-reported putative risk and resiliency factors in cadets starting the RCMP Cadet Training Program. Cadets’ scores were compared to published results from the Canadian, American, Australian, and European young adult population samples, as well as compared across sociodemographic variables within the cadet sample, which will provide detailed characterizations of a novel sample that facilitate future comparisons. Consistent with the pre-registered hypotheses from the RCMP Study Protocol (Carleton et al., 2022) and prior literature (Carleton et al., 2018; Korol et al., 2019; Angehrn et al., 2020), we hypothesized that RCMP cadets would report lower scores on putative risk variables and higher scores on resilience than the young adult populations, and women cadets would report higher scores on putative risk variables and lower scores on resilience than men cadets. Cadets who were married or common law, older, or who have completed higher education were expected to have lower scores on putative risk variables and higher scores on resilience (Afifi et al., 2006; Breslau et al., 2011; Carleton et al., 2018). By examining data from the Cadet Training Program, insight can be gleaned into cadets’ mental health prior to serving as RCMP officers.

## Materials and methods

### Procedure

Data were collected via an online Qualtrics self-report survey, available in both English and French, as part of the RCMP Longitudinal Study (Carleton et al., 2022). Data collection occurred between 22 April 2019 and 9 December 2019 and between 16 November 2020 and 3 October 2021; in the intervening interval, the RCMP Training Depot was closed due to the COVID-19 pandemic. Full details regarding the RCMP Study protocol (e.g., design, recruitment) are available in a dedicated protocol paper (Carleton et al., 2022). The RCMP Study was approved by both the University of Regina Institutional Research Ethics Board (File No. 2019-055) and the RCMP Research Ethics Board (File No. SKM_C30818021312580). The RCMP Study was also approved through a Privacy Impact Assessment as part of the overall approval by the National Administrative Records Management System 201611123286 and Public Services and Procurement Canada 201701491/M7594174191. The current study used data collected at the pre-training stage of the Cadet Training Program, a stage that included the initial assessment, pre-training survey, and clinical interview (Carleton et al., 2022). Data were statistically compared, using between-group analyses, to published young adult population norms for measures assessing anxiety sensitivity (Taylor et al., 2007), intolerance of uncertainty and illness and injury sensitivity (Fetzner et al., 2016), fear of negative evaluation (Hajdúk et al., 2015), pain-related anxiety (Abrams et al., 2007), state anger (Forbes et al., 2014), and resilience (Calo et al., 2019).

### Data and sample

Participation in the RCMP Study was voluntary. To enter the Cadet Training Program, recruits were required to be Canadian citizens or permanent residents, aged 19 to 57 years, and fluent in either English or French. Cadets also had to meet several recruitment requirements of the training program, including minimum physical standards, medical examinations, security clearance, a polygraph test, and some psychological testing (Hembroff and Krätzig, 2020). The current sample included 772 RCMP cadets, aged 19 to 52 years (*M* = 28.96, *SD* = 6.14), most of whom were men (72.2%) and White (70.7%). Following current best practices in the literature (Bauer et al., 2017; Lussenhop, 2018; Frohard-Dourlent et al., 2020), separate demographic questions were asked about participants’ gender identity and their sex. Some participants (*n* = 21; 2.7%) chose not to identify their gender. Among those who did answer, most were cisgender (99.5%), while 0.05% were transgender, nonbinary, or Two-Spirit. Due to confidentiality concerns for small groups, this paper reports on cisgender participants only.

Young adult population norms were obtained from several samples. Young adult norms were used as our cadet participants were generally in their early twenties, single, and with limited postsecondary education. Whenever possible, samples from the Canadian young adult populations were used, with samples from American, Australian, and European young adult populations used when obtaining Canadian published norms was not possible for a given measure. The anxiety sensitivity sample (Taylor et al., 2007) comprised 4,720 Canadian and American undergraduate students (66.8% women). The fear of negative evaluation sample (Hajdúk et al., 2015) comprised 332 European undergraduate students (74.1% female; M_age_ = 20.93), while the resiliency sample (Calo et al., 2019) consisted of 134 Australian senior physiotherapy students (55.2% women). The state anger sample (Forbes et al., 2014) consisted of 501 American undergraduate students (62.1% female; M_age_ = 19.58). The illness and injury sensitivity and intolerance of uncertainty (Fetzner et al., 2016) comprised 1,477 Canadian young adults (72.0% female; M_age_ = 25.59). The pain-anxiety sample (Abrams et al., 2007) comprised 155 Canadian undergraduate students (69.0% women; M_age_ = 20.41).

### Measures

#### Sociodemographic questions

RCMP cadets were asked to self-report their gender, sex, age, marital status, province of residence, highest education level attained, and whether they had prior PSP work experience.

#### Anxiety Sensitivity Index-3

Anxiety sensitivity is a dispositional fear of arousal-related sensations (e.g., increased heart rate, blushing; Taylor et al., 2007; Smits et al., 2008; Keough and Schmidt, 2012). The Anxiety Sensitivity Index-3 (ASI-3; Taylor et al., 2007) is an 18-item self-report measure used to assess anxiety sensitivity across three dimensions: somatic concerns (6 items; e.g., “It scares me when my heart beats rapidly”); cognitive concerns (6 items; e.g., “When I cannot keep my mind on a task, I worry that I might be going crazy”); and social concerns (6 items; e.g., “It is important for me not to appear nervous”). Participants rate items on a Likert scale from 0 (*agree very little*) to 4 (*agree very much*). Higher scores indicate greater sensitivity to anxiety symptoms. The ASI-3 has demonstrated strong internal consistency (Taylor et al., 2007) and good test–retest reliability (Osman et al., 2010), as well as good convergent, discriminant, and criterion validity (Taylor et al., 2007).

#### Brief Fear of Negative Evaluation Scale–Straightforward items

Fear of negative evaluation is dispositional apprehension experienced during evaluative situations (Rodebaugh et al., 2004; Weeks et al., 2005; Russell et al., 2021). The Brief Fear of Negative Evaluation Scale–Straightforward items (BFNE-S; Rodebaugh et al., 2004; Weeks et al., 2005) is an 8-item self-report measure used to assess fears of negative evaluation with the straightforward items from the original Brief Fear of Negative Evaluation Scale (Leary, 1983; Carleton et al., 2011). Participants rate each item (e.g., “I am usually worried about what kind of impression I make”) on a scale ranging from 0 (*not at all characteristic of me*) to 4 (*extremely characteristic of me*). Higher scores indicate greater fears of negative evaluation. The BFNE-S has demonstrated excellent internal consistency, construct validity, and factorial validity (Weeks et al., 2005; Rodebaugh et al., 2007; Carleton et al., 2007a).

#### Illness/Injury Sensitivity Index-Revised

Illness and injury sensitivity are dispositional tendencies to fear physical harm and injury (Carleton et al., 2005; Angehrn et al., 2020). The Illness/Injury Sensitivity Index-Revised (ISI-R; Carleton et al., 2005) is a 9-item measure revised from the original Illness/Injury Sensitivity Index (Taylor, 1993), and used to assess fear of illness (5 items; e.g., “I worry about becoming physically ill”) and fear of injury (4 items; e.g., “I am frightened of being injured”). Participants rate items using a scale ranging from 0 (*agree very little*) to 4 (*agree very much*). Higher scores indicate greater worries about illness and injury. Inter-correlation between the two factors is sufficient to justify use of a single total score (Carleton et al., 2006). The ISI-R has demonstrated excellent internal consistency and convergent validity with other measures pertaining to illness and injury (anxiety sensitivity somatic concerns, fear of pain, fear of movement, and re-injury), and high correlation with the original index (Carleton et al., 2006).

#### Intolerance of Uncertainty Scale-Short Form

Intolerance of uncertainty describes dispositional challenges that occur after perceiving the absence of salient, key, or sufficient information” (p. 31 Schmidt and Lerew, 1998; Schmidt et al., 1999; Carleton et al., 2007b). The Intolerance of Uncertainty Scale (IUS-12; Carleton et al., 2007b) is a 12-item self-report questionnaire used to assess difficulties tolerating uncertainty. Participants rate each item on a scale ranging from 1 (*not at all characteristic of me*) to 5 (*entirely characteristic of me*). The IUS-12 has two factors (Carleton et al., 2007b; McEvoy and Mahoney, 2012): prospective IU (7 items; e.g., “I cannot stand being taken by surprise”) and inhibitory intolerance of uncertainty (5 items; e.g., “I must get away from all uncertain situations). Higher scores indicate less ability to tolerate uncertainty, with the two factors correlating sufficiently to justify use of a single total score (Carleton et al., 2007b). The IUS-12 is strongly correlated with the original Intolerance of Uncertainty Scale and has demonstrated excellent convergent validity and internal consistency (Carleton et al., 2007b, 2012b).

#### Pain Anxiety Symptoms Scale-20

Pain-related anxiety is the dispositional tendency to respond to potential or actual pain with anxiety or fear (McCracken and Dhingra, 2002; Rogers et al., 2020). The Pain Anxiety Symptoms Scale-20 (PASS-20; McCracken and Dhingra, 2002) is a 20-item short form of the original Pain Anxiety Symptoms Scale (McCracken et al., 1992), used to assess pain-related anxiety across four dimensions: cognitive (5 items; e.g., “I cannot think straight when in pain”), fear (5 items; e.g., “Pain sensations are terrifying”), escape/avoidance (5 items; e.g., “I will stop any activity as soon as I sense pain coming on”), and physiological (5 items; e.g., “Pain makes me nauseous”). Participants rate items on a scale ranging from 0 (*never*) to 5 (*always*). Higher scores indicate greater anxiety about pain symptoms. The PASS-20 has demonstrated good factorial validity, for both the total scale and subscales, in both clinical (Coons et al., 2004) and non-clinical (Abrams et al., 2007) samples; strong internal consistency; and good construct and predictive validity (McCracken and Dhingra, 2002).

#### Dimensions of Anger Reactions

State anger is a putative risk factor for psychological distress following exposure to PPTE (Barsky and Ahern, 2004; Smith et al., 2008). The Dimensions of Anger Reactions (DAR-5; Barsky and Ahern, 2004) is a 5-item self-report questionnaire used to assess levels of anger. Participants rate each item (e.g., “When I got angry at someone, I wanted to hit them”) on a scale from 1 (*none or almost none of the time*) to 5 (*all or almost all of the time*). Higher scores indicate greater levels of anger. The DAR-5 has demonstrated strong internal reliability (Forbes et al., 2014) and concurrent validity with the State–Trait Anger Expression Inventory-2 (Tibubos et al., 2020).

#### Brief Resilience Scale

The Brief Resilience Scale (BRS; Smith et al., 2008) is a 6-item self-report questionnaire used to assess individual perceptions of adaptability to difficult situations. Participants rate each item (e.g., “I tend to bounce back quickly after hard times”) on a scale ranging from 1 (*strongly disagree*) to 5 (*strongly agree*; Smith et al., 2008). Higher scores indicate greater individual resilience. The BRS has demonstrated good internal consistency and test–retest reliability (Windle et al., 2011) and convergent and divergent validity (Sánchez et al., 2021).

### Statistical analyses

Some participants completed the Cadet Training Program prior to the closure of RCMP Training Depot due to the COVID-19 pandemic, and others completed the Cadet Training Program after RCMP Training Depot reopened. Differences across putative risk and resiliency variables between participants completing the Cadet Training Program pre- and post-COVID-19 were assessed using independent samples *t*-tests. Descriptive statistics were calculated for each putative risk and resiliency factor. To test the hypothesis that cadets would report lower scores on putative risk variables and higher scores on resilience compared to young adult samples, one-sample *t*-tests were used to compare cadets’ scores to published scores from samples of Canadian, American, Australian, and European young adult populations (Abrams et al., 2007; Taylor et al., 2007; Forbes et al., 2014; Hajdúk et al., 2015; Fetzner et al., 2016; Calo et al., 2019), with Cohen’s *d* used to measure effect size. To test whether men and male cadets and cadets who are married or common law, older, or who have completed higher education reported lower scores on risk variables and higher scores on resilience, independent samples *t*-tests and one-way ANOVAs were conducted with the cadet data. Holm-Bonferroni adjustments were applied to alpha levels in *post hoc* analyses to control for Type I errors in multiple comparisons.

## Results

### Comparison of cadet scores with the young adult population norms

Table 1 provides descriptive statistics and a summary of analyses comparing putative risk and resiliency variables between cadets and the young adult populations. Cadets had statistically significantly lower scores on all putative risk variables examined [all *p*s < 0.001, *d* = 0.239 (anxiety sensitivity social concerns)—1.881 (state anger)] and statistically significantly higher BRS scores compared to the young adult population (*p* < 0.001, *d* = 0.873).

**Table 1:** Putative risk and resilience factor between-group differences among cadets and the general population.

	Cadets				General population	Test statistics	
Variable	Mean (*SD*)	α	Skew (SE = 0.11)	Kurtosis (SE = 0.21)	Mean (*SD*)	*t*	Effect size (Cohen’s *d*)
Anxiety sensitivity—global	8.30 (8.22)	0.89	2.06	5.26	12.80 (10.60)	15.44^***^	0.548
Anxiety sensitivity—somatic	1.73 (2.88)	0.84	2.52	7.67	4.20 (4.42)	25.94^***^	0.937
Anxiety sensitivity—cognitive	1.59 (2.82)	0.85	2.70	8.09	2.70 (3.80)	10.95^***^	0.395
Anxiety sensitivity—Social	4.99 (3.81)	0.71	1.11	1.04	5.90 (4.70)	6.62^***^	0.239
Fear of negative evaluation	16.96 (7.52)	0.96	0.72	−0.11	21.95 (8.72)	18.22^***^	0.663
Illness and injury sensitivity—global	7.40 (7.61)	0.93	1.17	0.82	12.82 (9.84)	19.76^***^	0.711
Illness sensitivity	3.81 (4.41)	0.90	1.32	1.27	6.14 (5.32)	14.64^***^	0.527
Injury sensitivity	3.59 (3.70)	0.88	1.06	0.46	8.26 (6.23)	35.06^***^	1.263
Intolerance of uncertainty—global	21.75 (7.06)	0.88	0.83	0.69	31.23 (11.61)	37.11^***^	1.343
Intolerance of uncertainty—inhibitory	7.10 (2.74)	0.77	1.64	2.94	11.91 (5.54)	48.52^***^	1.756
Intolerance of uncertainty—prospective	14.65 (4.99)	0.81	0.48	−0.06	19.32 (6.80)	25.89^***^	0.937
Pain anxiety sensitivity—global	11.75 (11.91)	0.92	1.37	1.74	24.04 (13.45)	28.59^***^	1.032
Pain anxiety sensitivity—cognitive	4.43 (4.46)	0.91	1.12	0.81	9.04 (5.22)	28.67^***^	1.034
Pain anxiety sensitivity—escape/avoidance	4.14 (4.20)	0.81	1.32	1.90	6.37 (3.82)	14.70^***^	0.530
Pain anxiety sensitivity—fear	2.08 (3.48)	0.86	2.08	4.34	4.05 (3.67)	15.65^***^	0.565
Pain anxiety sensitivity—physiological arousal	1.10 (2.28)	0.83	2.51	6.52	4.59 (4.04)	42.44^***^	1.531
State anger	5.92 (1.69)	0.71	3.69	21.08	9.10 (4.00)	52.28^***^	1.881
Resilience	3.96 (0.60)	0.77	−0.17	−0.09	3.43 (0.76)	24.10^***^	0.873

Anxiety sensitivity—global = ASI-3 total score; Anxiety sensitivity—somatic = ASI-3 somatic concerns; Anxiety sensitivity—cognitive = ASI-3 cognitive concerns; Anxiety sensitivity—social = ASI-3 social concerns; Fear of negative evaluation = BFNES; Resilience = BRS; Anger = DAR-5; Illness and injury sensitivity—global = ISI-R total score; Illness sensitivity = ISI-R illness; Injury sensitivity = ISI-R injury; Intolerance of uncertainty—Global = IUS-12 total score; Intolerance of uncertainty—inhibitory = IUS-12 inhibitory concerns; Intolerance of uncertainty—prospective = IUS-12 prospective concerns; Pain anxiety sensitivity—Global = PASS-20 total score; Pain anxiety sensitivity—cognitive = PASS-20 cognitive dimension; Pain Anxiety sensitivity—escape/avoidance = PASS-20 escape/avoidance dimension; Pain anxiety sensitivity—fear = PASS-20 fear dimension; pain anxiety sensitivity—physical = PASS-20 physiological arousal dimension. ^*^p ≤ 0.05; ^**^p ≤ 0.01; ***p ≤ 0.001—statistically significant after Holm-Bonferroni correction.

### Cadet risk and resiliency scores

There were no statistically significant differences across putative risk and resiliency variables between participants who completed the Cadet Training Program pre- and post-COVID-19. Therefore, pre- and post-COVID-19 participants were amalgamated, and further analyses were conducted on the amalgamated sample.

All putative risk variables were statistically significantly positively associated with one another. Resilience was statistically significantly negatively associated with all putative risk variables, except for ISI-R and IUS-12, with which resilience was statistically significantly positively associated. Table 2 provides results from between-group analyses of putative risk and resiliency variables across sociodemographic groups. Gender and sex comparisons were made based on available sample sizes with resulting difference patterns being relatively comparable. Women, *t*(731) = 4.05, *p* < 0.001, *d* = 0.342, and female, *t*(734) = 3.74, *p* < 0.001, *d* = 0.314, cadets had significantly lower BRS scores than men and male cadets.

**Table 2:** Sociodemographic characteristics and comparison of putative risk and resiliency outcomes scores among different categories.

	%(*n*)^1^	Anxiety sensitivity—global	Fear of negative evaluation	Resilience	State anger	Illness and injury sensitivity	Intolerance of uncertainty	Pain anxiety sensitivity	Mean (*SD*)	Mean (*SD*)	Mean (*SD*)	Mean (*SD*)	Mean (*SD*)	Mean (*SD*)	Mean (*SD*)
**Gender**								
Man	72.2 (557)	8.22 (8.40)	16.74 (7.48)	4.00 (0.60)	5.85 (1.68)	7.30 (7.69)	21.50 (6.95)	10.67 (11.34)
Woman	24.6 (190)	8.48 (7.70)	17.49 (7.50)	3.80 (0.60)	6.01 (1.55)	7.61 (7.47)	22.16 (7.21)	14.86 (12.97)
Effect size (Cohen’s *d*)		0.031	0.101	0.342^***^	0.097	0.041	0.095	0.356^***^
**Sex**								
Male	72.0 (556)	8.27 (8.46)	16.81 (7.53)	4.00 (0.60)	5.85 (1.68)	7.35 (7.72)	21.55 (7.03)	10.73 (11.42)
Female	25.1 (194)	8.43 (7.64)	17.43 (7.49)	3.81 (0.61)	6.03 (1.56)	7.52 (7.42)	22.14 (7.17)	14.69 (12.90)
Effect size (Cohen’s *d*)		0.019	0.083	0.314***	0.110	0.022	0.083	0.335^***^
**Age**								
19–29	59.8 (462)	8.30 (8.05)	17.50 (7.55)	3.94 (0.60)	5.88 (1.68)	7.59 (7.67)	21.61 (7.11)	11.93 (11.37)
30–39	28.0 (216)	8.55 (8.65)	16.48 (7.57)	3.96 (0.60)	6.03 (1.76)	7.20 (7.33)	22.41 (7.19)	11.73 (12.64)
40–49	6.3 (49)	7.57 (8.70)	14.34 (6.51)	4.08 (0.62)	5.70 (0.86)	5.21 (7.06)	19.87 (5.36)	10.11 (12.53)
50–59	0.6 (5)	5.80 (5.97)	16.00 (7.38)	3.97 (0.49)	5.40 (0.55)	9.80 (10.26)	22.20 (5.76)	14.60 (18.49)
Effect size ( $\eta_{p}^{2}$ )		0.001	0.012^*^	0.003	0.003	0.007	0.008	0.002
**Marital status**								
Single	47.2 (364)	8.59 (8.65)	17.51 (7.84)	3.90 (0.61)	5.86 (1.59)	7.77 (7.92)	22.23 (7.78)	12.57 (12.12)
Separated/divorced	1.6 (12)	10.27 (6.23)	20.09 (5.75)	3.95 (0.40)	5.75 (0.75)	11.40 (8.97)	26.09 (6.92)	18.64 (12.48)
Married/common-law	42.9 (331)	8.11 (7.93)	16.16 (7.20)	4.01 (0.59)	5.93 (1.74)	6.94 (7.13)	21.21 (6.19)	10.94 (11.87)
Widowed	0 (0)	0 (0)	0 (0)	0 (0)	0 (0)	0 (0)	0 (0)	0 (0)
Effect size ( $\eta_{p}^{2}$ )		0.002	0.011*	0.009*	0.001	0.007	0.011^*^	0.009^*^
**Province of residence**								
Western Canada (BC, AB, SK, MB)	52.8 (408)	8.21 (7.67)	17.30 (7.54)	3.97 (0.59)	6.08 (1.88)	8.04 (7.72)	22.12 (6.83)	12.13 (12.43)
Eastern Canada (ON, QC)	34.6 (267)	8.37 (9.08)	16.53 (7.45)	3.96 (0.62)	5.70 (1.44)	6.61 (7.42)	21.26 (7.33)	11.44 (11.78)
Atlantic Canada (PEI, NS, NB, NFL)	11.3 (87)	8.33 (8.16)	16.59 (7.64)	3.90 (0.63)	5.86 (1.48)	7.15 (7.88)	21.51 (7.29)	11.38 (10.49)
Northern Territories (YK, NWT, NVT)	1.0 (8)	9.63 (7.67)	18.14 (8.03)	3.85 (0.57)	5.25 (0.46)	4.50 (4.31)	19.50 (7.78)	10.13 (6.36)
Effect size ( $\eta_{p}^{2}$ )		0.000	0.003	0.002	0.012*	0.009	0.004	0.001
**Education**								
High school graduate or less	10.2 (79)	8.91 (7.92)	16.13 (5.72)	3.92 (0.60)	6.22 (2.56)	7.95 (7.43)	22.32 (6.82)	12.66 (12.13)
Some post-secondary school	43.4 (335)	8.16 (8.18)	17.14 (7.85)	3.98 (0.58)	5.87 (1.53)	7.28 (7.51)	21.42 (7.07)	11.31 (11.16)
University degree/4-year college or higher	39.5 (305)	8.33 (8.42)	17.02 (7.64)	3.92 (0.62)	5.84 (1.41)	7.44 (7.90)	21.89 (7.17)	12.13 (13.01)
Effect size ( $\eta_{p}^{2}$ )		0.001	0.002	0.002	0.005	0.001	0.002	0.002
**Prior PSP service**								
Yes	30.7 (237)	7.35 (7.35)	16.33 (7.36)	4.01 (0.56)	6.02 (1.94)	7.08 (7.51)	21.15 (6.73)	10.60 (11.27)
No	60.1 (464)	8.54 (8.43)	17.16 (7.68)	3.95 (0.63)	5.87 (1.61)	7.35 (7.69)	21.87 (7.26)	11.92 (12.18)
Effect size (Cohen’s d)		0.147	0.108	0.102	0.086	0.036	0.101	0.111

Anxiety sensitivity = ASI-3 Total score; Fear of negative evaluation = BFNE-S; Resilience = BRS; Anger = DAR-5; Illness and injury sensitivity = ISI-R total score; Intolerance of uncertainty = IUS-12 Total score; Pain anxiety sensitivity = PASS-20 total score. ^*^p ≤ 0.05; ^**^p ≤ 0.01; ^***^p ≤ 0.001—Statistically significantly different; Holm-Bonferroni adjustment applied to alpha levels to control Type I error. M (SD) represents Mean (Standard Deviation). $\eta_{p}^{2}$ represents partial Eta Square. Lettered superscripts within each column category indicate significant differences between category groups on respective screening measure at *p* ≤ 0.05. Means followed by a common letter are not significantly different.

1 Total percentages may not sum to 100 and ns may not sum to 772 due to non-response or responding “other.”

A one-way ANOVA evidenced a statistically significant effect of age on BFNE-S scores, *F*(3, 706) = 2.88, *p* = 0.035, $\eta_{p}^{2}$ = 0.012. A *post-hoc* Tukey test evidenced that cadets 19–29 years of age had statistically significantly higher scores on the BFNE-S than cadets 40–49 years of age (*p* = 0.037); however, the difference was no longer statistically significant following a Holm-Bonferroni correction. Age groupings were based on previous publications (Carleton et al., 2018, 2020). Results of several one-way ANOVAs indicated statistically significant differences across marital status groups on the BFNE-S, *F*(2, 682) = 3.70, *p* = 0.025, $\eta_{p}^{2}$ = 0.011; the BRS, *F*(2, 691) = 3.31, *p* = 0.037, $\eta_{p}^{2}$ = 0.009; the IUS-12, *F*(2, 690) = 3.82, *p* = 0.022, $\eta_{p}^{2}$ = 0.011; and the PASS-20, *F*(2, 695) = 3.32, *p* = 0.037, $\eta_{p}^{2}$ = 0.009 (see Table 2). *Post-hoc* Tukey tests showed that for the BFNE-S, differences were only statistically significant (*p* = 0.043) between cadets who were single and married or common law, with those who were single having statistically significantly higher scores. Similarly, differences on resiliency scores were only statistically significant (*p* = 0.03) between cadets who were single and married or common law, with those who were single having statistically significantly lower scores. For both the BFNE-S and the BRS, however, the differences were no longer statistically significant following Holm-Bonferroni corrections. Finally, a one-way ANOVA indicated that there were statistically significant differences across the province of residence in DAR-5 reactivity, *F*(3, 761) = 3.16, *p* = 0.024, $\eta_{p}^{2}$ = 0.012. A *post-hoc* Tukey test showed that only cadets from Western and Eastern Canada differed significantly (*p* = 0.026), with those from Western Canada having statistically significantly higher scores; however, the difference was no longer statistically significant following a Holm-Bonferroni correction. There were no statistically significant effects of education group or of prior PSP service.

## Discussion

The results were consistent with the current pre-registered hypotheses that were also clarified in the protocol paper (Carleton et al., 2022); specifically, RCMP cadets at pre-training reported statistically significantly and substantially lower scores on all putative risk variables and higher scores on resilience than the young adult populations. The current results indicate that, as a function of the RCMP selection processes, self-selection biases among persons who apply to become RCMP, and preparation for attending the Cadet Training Program, RCMP cadets at pre-training may be at substantially reduced risk for mental health challenges (Carleton et al., 2022).

The current results directly contrast the notion that the higher prevalence of mental health challenges in RCMP officers is related to inherent psychological vulnerabilities or insufficiencies that can be screened out as part of recruitment (Horswill and Carleton, 2021) to mitigate the high prevalence of mental health disorders among serving RCMP (Carleton et al., 2018). Consistent with the extant literature, the higher prevalence of positive screening for mental health challenges among serving RCMP (i.e., 50.2%) relative to the general population (i.e., 10.1%; Government of Canada, 2012; Carleton et al., 2018) may be associated with frequent PPTE exposures (Galatzer-Levy et al., 2011; Kilpatrick et al., 2013; Carleton et al., 2019) and other occupational stressors (Pozzulo et al., 2017; Carleton et al., 2020; Ricciardelli et al., 2020b) or the interaction of individual vulnerabilities with occupational stressors (Schmidt et al., 1997). Cadets who have relatively higher scores compared to other cadets may still be at risk of experiencing mental health challenges due to interactions between innate susceptibility and environmental susceptibility (i.e., exposure to PPTE). Army recruits undergoing basic training who had significantly lower AS scores compared to the general population, but relatively higher scores compared to fellow recruits, were more likely than other recruits to experience panic attacks during basic training (Schmidt et al., 1997). The subsequent RCMP Study data collections will allow for testing of the posited causal and interactive relationships between dispositional risk and resilience variables, diverse stressors, coping activities which may also serve as risk or resilience factors, and mental health. In the interim, the current results do not indicate a need for specific changes to recruitment screening as part of efforts to mitigate subsequent mental health challenges; instead, the current results are consistent with the extant literature and suggest that ongoing evidence-based training, assessment, and treatment, as well as pervasive organizational supports may be required to protect RCMP officers from adverse mental health outcomes during their career (Stelnicki et al., 2021; Carleton et al., 2022).

Among cadets, and consistent with the research literature (Galatzer-Levy et al., 2011; Kilpatrick et al., 2013; Carleton et al., 2020), women cadets scored lower on resilience than men cadets (Galatzer-Levy et al., 2011; Kilpatrick et al., 2013; Carleton et al., 2020). This difference may be attributable to a range of individual- and structural-level factors. Research shows that women tend to underestimate and underreport their own abilities (e.g., Vajapey et al., 2020; Reilly and Andrews et al., 2022), which could affect self-assessments of resilience. Coping strategies, which are linked to resilience, may be gendered (Ménard and Arter, 2014). Police women’s resilience may also be affected by structural factors related to gender inequality, such as women’s disproportionate responsibility for unwaged and caregiving work (Moyser and Burlock, 2018); different access to social supports (Violanti et al., 2016; Kaur et al., 2021); gender-based harassment, discrimination, and stereotyping of women in men-dominated fields like policing (Angehrn et al., 2021a); or relative differences in control over work (Violanti, 1992) and environmental mastery (i.e., being able to intentionally manipulate the surrounding context and events; Boardman et al., 2008). Additional qualitative research (e.g., interviews, focus groups) with women cadets is required to better understand what appears to be a gender-based difference in resilience.

The current study had several strengths and limitations. Strengths included: (1) the large sample size with inclusion of both pre- and during-COVID subsamples allowed results to be generalized to both pandemic and non-pandemic circumstances and (2) the array of established mental health risk factors assessed for the first time with RCMP cadets at pre-training. There were also several limitations. First, every effort was made to find normative results for putative risk and resiliency variables from the Canadian young adult population; however, not all results were available, so for some variables American, Australian, and European normative results were used. Most participants in the young adult samples were women, whereas most of the RCMP cadets were men; nevertheless, there were few gender differences on the putative risk and resiliency variables among cadets, suggesting effects from the uncontrolled variables would be small. Second, the cross-sectional nature of the data did not allow for causal conclusions concerning job demands and RCMP officers’ mental health. Further data collection in the ongoing and longitudinal RCMP Study will allow for an examination of causal relationships with mental health variables, and interaction effects from PPTE exposures and other occupational stressors. Third, the current study did not include serving RCMP officers or an in-depth assessment of sex or gender differences across cadets and officers. Such analyses are necessary for a more robust understanding of the putative risk and resiliency factors among RCMP officers. Fourth, there is a potential for socially desirable responding, which is likely mitigated by emphasis on the confidentiality, anonymity, and independence of the study from the RCMP.

Future researchers should assess for differences in putative risk and resiliency variables between RCMP cadets, RCMP officers, and the general population, and include a robust examination of sex and gender differences. The extant literature suggests that there are differences among male and female police officers’ mental health symptom reporting (Carleton et al., 2018; Angehrn et al., 2021b). Future research using the developing RCMP dataset should also assess for changes in putative risk factors and resilience over time, as well as interactions with sociodemographic characteristics (e.g., gender, age, service location), training, occupational stressors, and mental health. Research using matched participants may also increase the robustness of the conclusions that can be drawn. The associated results may provide important information on how best to protect RCMP member mental health using a variety of tools. Future research with the developing RCMP dataset should also investigate opportunities to protect against the negative effects of service on RCMP members, such as the impact of the Emotional Resilience Skills Training, a 13-week protocol based on the Unified Protocol (Carleton et al., 2022).

### Conclusion

The current study was the first to assess mental health risk and resiliency factors in a sample of RCMP cadets beginning their training. The results indicate that RCMP cadets start training with lower scores on several putative risk variables and higher scores on resiliency, relative to normative scores on these measures in the general population. This may suggest that RCMP cadets are actually *less* vulnerable to developing mental health challenges as compared to the general population. Accordingly, previously documented *elevated* prevalence of mental health disorders in RCMP officers compared to the general population is likely associated with occupational stressors associated with their service or interactions of service-related stressors with premorbid vulnerability. The current results contraindicate new screening tools and instead favor ongoing evidence-based training, assessment, and treatment, as well as pervasive organizational supports as best strategies for protecting RCMP members’ mental health; however, that supposition will be tested with subsequent results from the developing RCMP Study dataset as longitudinal data come in over time.

## Data availability statement

The datasets presented in this article are not readily available because the datasets will be made available only for independent confirmation purposes and only to persons with the necessary ethical and security clearances as defined by the research ethics board at the University of Regina and the contractual obligations with the Royal Canadian Mounted Police. Requests regarding the datasets can be made to the corresponding author. Requests to access the datasets should be directed to RNC, nick.carleton@uregina.ca.

## Ethics statement

Data for the current paper were collected as a part of the broader RCMP Study. The associated protocol paper provides full details of the RCMP longitudinal Study (Carleton et al., 2022). The RCMP Study was approved by the University of Regina Ethics Board on April 10, 2019 (File #2019-055), and the RCMP Research Ethics Board followed with approval on April 12, 2019 (File #SKM_C30818021312580). The study was also approved through a Privacy Impact Assessment as part of the overall National Administrative Records Management System approval (201611123286) and Public Services and Procurement Canada approval (201701491/M7594174191). The project is bound by the Privacy Act, R.S., 1985, c. P-21 and the Personal Information Protection and Electronic Documents Act, SC. 2000, c.5 and approved by Public Services and Procurement Canada (PSPC) M7594-171491/001/SS. The participants provided their electronically-recorded informed consent to participate in this study.

## Author contributions

RNC, LJ, and JK: conceptualization, methodology, and formal analysis. GA, RNC, LJ, JK, and SS: validation. RNC: investigation, resources, project administration, and funding acquisition. RNC, LJ, JK, JN, and RS: writing—original draft preparation. JK, LJ, RS, JN, TA, AF, SS, GA, SS-Z, GK, and RNC: writing—review and editing. RNC and LJ: supervision. All authors contributed to the article and approved the submitted version.

## Funding

RNC was supported by a Medavie Foundation Project Grant. TA was supported by a Tier I Canada Research Chair in Childhood Adversity and Resilience. SS was supported by a Tier 1 Canada Research Chair in Addictions and Mental Health. The current study was supported by the RCMP, the Government of Canada, and the Ministry of Public Safety and Emergency Preparedness.

## Conflict of interest

The authors declare that the research was conducted in the absence of any commercial or financial relationships that could be construed as a potential conflict of interest.

## Publisher’s note

All claims expressed in this article are solely those of the authors and do not necessarily represent those of their affiliated organizations, or those of the publisher, the editors and the reviewers. Any product that may be evaluated in this article, or claim that may be made by its manufacturer, is not guaranteed or endorsed by the publisher.
